# Isolated Arterial Hypertension as a Rare Early Manifestation of Guillain-Barré Syndrome: A Case Report

**DOI:** 10.7759/cureus.106941

**Published:** 2026-04-13

**Authors:** El Moufid Hajjar, Widade Kojmane

**Affiliations:** 1 Pediatric Emergency Department, Centre Hospitalier Universitaire Hassan II, Fès, MAR

**Keywords:** arterial hypertension, child, dysautonomia, flaccid paralysis, guillain-barré syndrome

## Abstract

Guillain-Barré syndrome (GBS) is a well-known post-infectious acute polyradiculoneuritis, classically characterized by ascending muscle weakness. While dysautonomia affects up to two-thirds of patients, it almost always follows the onset of motor deficits. Arterial hypertension, when presenting as an isolated premonitory sign occurring weeks before neurological involvement, constitutes an exceptional clinical pitfall that can lead to exhaustive and unnecessary etiological assessments. We report the case of a 9-year-old girl with no prior medical history, initially admitted for persistent headaches, vomiting, and severe arterial hypertension (160/110 mmHg). An extensive initial investigation for secondary hypertension (renal Doppler, echocardiography, catecholamine levels, cortisol, and renin-aldosterone system) was normal. It was only 20 days later, with the onset of facial paralysis and progressive limb weakness (3/5 strength), that the diagnosis was reconsidered. Electromyography confirmed an axonal-demyelinating sensory-motor polyneuropathy, and cerebrospinal fluid analysis revealed classic albuminocytologic dissociation. The patient was treated with intravenous immunoglobulins, leading to favorable motor recovery and complete normalization of blood pressure. This observation illustrates a highly unusual "hypertension-first" presentation of GBS. It serves as an essential reminder for pediatricians: unexplained acute arterial hypertension can be the sole inaugural manifestation of GBS-related dysautonomia. Recognizing this early signal is crucial to avoid diagnostic delays and initiate life-saving treatment before the onset of severe respiratory or motor failure.

## Introduction

Guillain-Barré syndrome (GBS) is an acute autoimmune peripheral neuropathy, classically characterized by the onset of acute ascending flaccid paralysis. Dysautonomic signs, particularly arterial hypertension, most often occur after the onset of motor deficit. However, it is exceptional for these manifestations to precede muscle weakness.

## Case presentation

A 9-year-old previously healthy girl, with up-to-date immunizations, presented with afebrile diffuse headaches and vomiting 20 days prior to the onset of motor deficits. Initial evaluation revealed severe arterial hypertension (160/110 mmHg). Following an unremarkable initial brain scan, a comprehensive etiological workup for secondary hypertension was initiated, focusing on endocrine and renal function. An initial plain radiograph of the chest and abdomen showed no abnormalities. Furthermore, systematic clinical and laboratory investigations ruled out structural renal abnormalities, adrenal masses, and renal artery stenosis as potential causes for the severe hypertension. The absence of identifiable secondary mechanical or endocrine triggers prompted further neurological and specialized laboratory investigations.

Upon the subsequent development of functional motor impairment, she was transferred to the pediatric emergency department. At admission, the patient was fully alert (Glasgow Coma Scale 15) and afebrile. While on antihypertensive medication, her blood pressure was 130/108 mmHg with a heart rate of 120 bpm. She remained respiratory stable without swallowing difficulties, though she exhibited mild peripheral facial nerve palsy. Neurological examination highlighted symmetric quadriparesis with a muscle strength of 3/5 in all four extremities and generalized areflexia, alongside fully preserved sensory function. A routine ophthalmological examination was unremarkable.

Diagnostic investigations showed negative inflammatory and infectious screens. An extensive biological and laboratory workup was performed to rule out secondary causes of hypertension and confirm the diagnosis. The results, including normal endocrine markers and characteristic cerebrospinal fluid (CSF) findings, are summarized in Table 1.

**Table 1:** Summary of the patient's laboratory and biological workup Urinary catecholamines were deemed clinically non-significant for a secreting tumor despite being at the upper limit of normal. CSF: cerebrospinal fluid

Laboratory parameter	Patient's result	Reference range
24-hour proteinuria	131 mg/24h	<150 mg/24h
Urinary adrenaline	0.11 µmol/24h	<0.10 µmol/24h
Urinary noradrenaline	0.60 µmol/24h	<0.50 µmol/24h
Serum cortisol (8:00 AM)	Normal	5-25 µg/dL
Serum aldosterone	Normal	3-35 ng/dL
Plasma renin activity	Normal	0.2-3.3 ng/mL/h
Complement C3	1.50 g/L	0.90-1.80 g/L
Complement C4	0.40 g/L	0.10-0.40 g/L
CSF protein level	Elevated	15-45 mg/dL
CSF white blood cell count	3 cells/mm³	<5 cells/mm³

**Figure 1:**
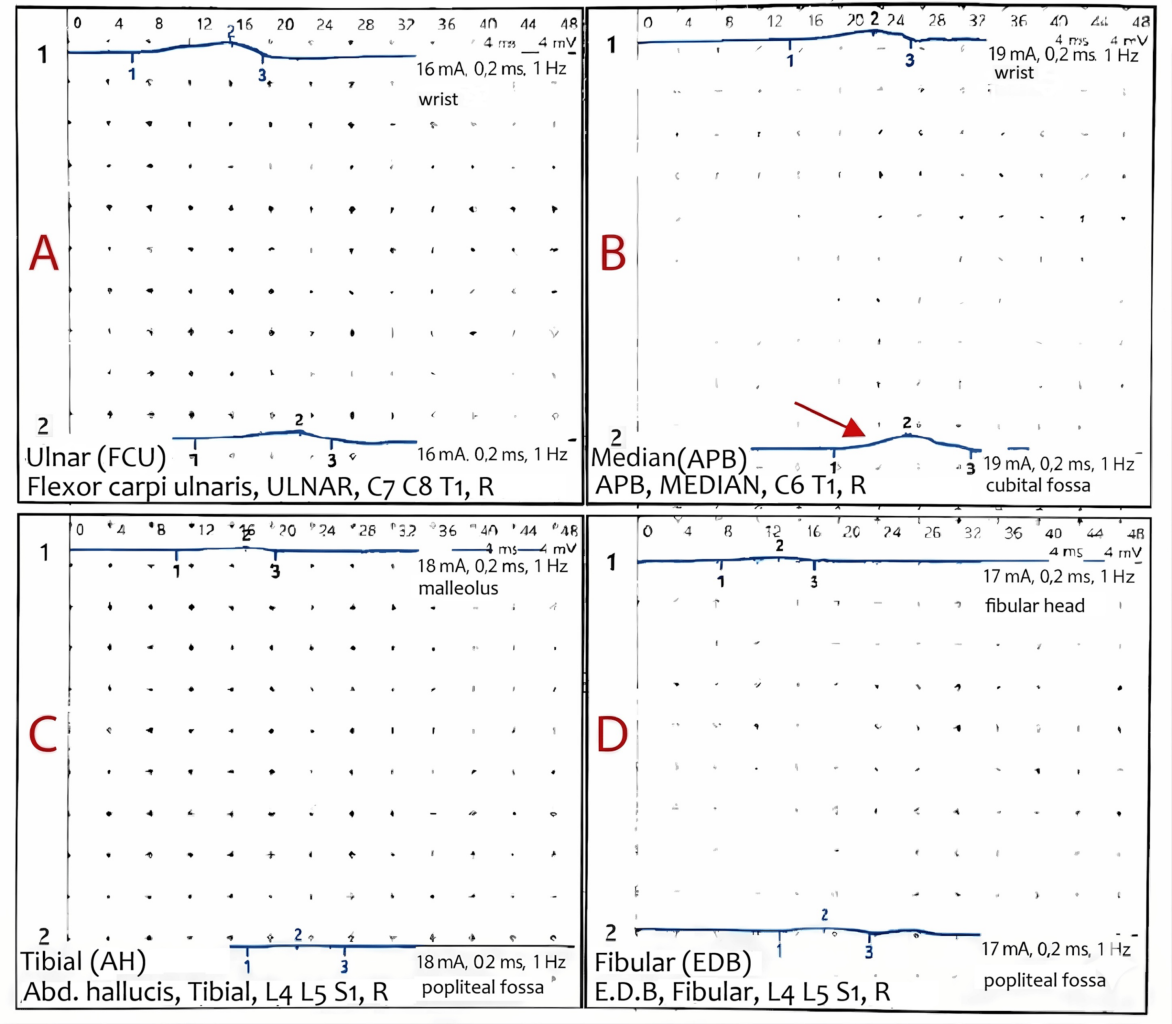
Multichannel EMG motor conduction study. The waveforms demonstrate prolonged distal motor latencies across the tested nerves A: ulnar, B: median, C: tibial, D: fibular EMG: electromyography Pane B illustrates the markedly delayed response of the right median nerve, indicated by the red arrow.

Electromyography (EMG) and nerve conduction studies established the diagnosis of an axonal-demyelinating sensorimotor polyneuropathy. The quantitative analysis revealed a marked increase in distal motor latencies, most notably reaching 15.0 ms for the right median nerve, along with severely reduced compound muscle action potential amplitudes and a complete absence of sensory responses in both the median (Digit II) and ulnar (Digit V) nerves. These markedly delayed and dispersed motor conduction waveforms are illustrated in Figure 1, where Pane B specifically highlights the pronounced pathological delay in the median nerve.

Comprehensive viral serologies, as well as systemic and immunological panels, were negative. The patient was treated with intravenous immunoglobulins (IVIg) at 1 g/kg/day for two consecutive days. She exhibited a favorable clinical trajectory, achieving complete normalization of her blood pressure alongside a partial recovery of her motor function. She was subsequently discharged to continue a structured outpatient motor rehabilitation program.

## Discussion

GBS is the most common cause of acute or subacute ascending flaccid paralysis, with an estimated global incidence between 0.4 and 1.7 cases per 100,000 per year, affecting children and adults of all ages without sex predominance [1]. The typical clinical manifestation is progressive ascending muscle weakness, accompanied by variable sensory loss occurring over several days to weeks, which can lead to respiratory failure in severe forms.

The clinical picture is generally reached within four weeks. The diagnosis of GBS is based on a cluster of clinical, biological, and electrophysiological arguments. CSF analysis shows albuminocytologic dissociation (an elevation in CSF protein levels associated with a normal cell count), which may be absent during the first few days. The EMG confirms the diagnosis and allows for differentiation between axonal and demyelinating forms of the disease. GBS is a heterogeneous group of acute autoimmune neuropathies, classified according to immunopathology and clinical presentation. It includes acute inflammatory demyelinating polyradiculoneuropathy, the most frequent form, as well as axonal forms, such as acute motor axonal neuropathy and acute motor and sensory axonal neuropathy. Added to these peripheral forms are several clinical variants belonging to the same spectrum, notably Miller Fisher syndrome, Bickerstaff brainstem encephalitis, acute pandysautonomia, pure sensory neuropathies, and pure motor neuropathies. All these entities share an autoimmune mechanism, most often occur in a post-infectious context, and evolve acutely, with an aggravation phase not exceeding four weeks [2].

Dysautonomia during GBS corresponds to involvement of the autonomic nervous system and occurs in approximately 40% to 66% of patients [3]. It manifests through a wide variety of functional disorders, notably blood pressure fluctuations; sinus tachycardia or bradycardia; abnormal sweating, such as anhidrosis or diaphoresis; and vasomotor flushing, as well as visceral disorders such as intestinal ileus or urinary retention. Pathophysiologically, these manifestations result from the involvement of autonomic pathways, with dysregulation of cardiovascular and visceral control mechanisms, including afferent arterial baroreceptors, efferent cardiac parasympathetic fibers, and preganglionic sympathetic fibers responsible for vasomotor and sudomotor functions [3]. Another pathophysiological hypothesis proposes that the dysautonomia observed in GBS results from a massive release of catecholamines secondary to a conduction block of peripheral afferent nerve fibers. These manifestations are generally transient, but their exact duration remains poorly determined. Furthermore, several studies indicate that subclinical indices of autonomic nervous system dysfunction can persist, as detected by biological analysis, even after clinical resolution of the syndrome [4].

Finally, research, notably by Chakraborty et al., has shown that GBS patients presenting with dysautonomia have a more unfavorable prognosis than those who do not, with prolonged hospitalization, less favorable functional outcomes at discharge, and a higher mortality rate (6% to 13% compared to approximately 2%) [5]. According to the study by Pagaling et al., the most frequent manifestation of dysautonomia affects the cardiovascular system, contributing to significant morbidity and mortality in GBS patients [6]. Although the exact mechanism remains incompletely elucidated, some observations suggest that GBS subtypes differ in their dysautonomic profiles: acute inflammatory demyelinating syndrome is characterized by cardiac sympathetic hyperactivity, whereas acute inflammatory axonal syndrome is associated with decreased sudomotor function, particularly in severe cases [7]. The pathophysiology of dysautonomia mainly involves a sympathetic imbalance, affecting afferent arterial baroreceptors, efferent cardiac parasympathetic fibers, and preganglionic sympathetic fibers [3]. Cardiovascular collapse can occur due to significant fluctuations in blood pressure, related to impaired feedback control or ectopic discharges following demyelination of preganglionic sympathetic axons or axonal degeneration of postganglionic axons [5]. These hypotheses are supported by histopathological studies revealing demyelination and mononuclear cell infiltration in the vagus nerve and sympathetic ganglia of patients who died from GBS [8].

Cardiovascular and autonomic disorders associated with GBS generally occur in patients with diffuse motor involvement and can manifest both during the aggravation phase and the plateau phase. These disorders are considered indicators of poor prognosis [9,10]. Table 2 summarizes the stark contrast between typical dysautonomic manifestations in GBS reported in the literature and the atypical presentation observed in our patient.

**Table 2:** Comparison of typical dysautonomic features in GBS versus the atypical presentation in our pediatric case GBS: Guillain-Barré syndrome

Clinical feature	Typical GBS dysautonomia (literature)	Our patient's presentation
Prevalence	Occurs in 40-66% of patients [3].	Present.
Time of onset	Generally, during the aggravation or plateau phase, there is diffuse motor involvement.	20 days prior to the onset of acute ascending flaccid muscle weakness.
Manifestations	Polymorphic: blood pressure fluctuations, tachycardia/bradycardia, sweating abnormalities, intestinal ileus, urinary retention [3].	Isolated acute arterial hypertension (revealed by signs of intracranial hypertension).
Prognostic value	Unfavorable prognosis, higher mortality rate, prolonged hospitalization [5,9,10].	Favorable outcome, clear improvement with IVIg, and complete normalization of blood pressure.

In our observation, hypertension began 20 days before the onset of muscle weakness and persisted until the recovery phase. However, the patient responded favorably to treatment, with a clear improvement in muscle strength, no neurological sequelae, and normalization of blood pressure. Therapeutic recommendations for GBS include IVIg and plasmapheresis, both of which are effective. In our patient, immunoglobulins at a dose of 1 g/kg/day for two days were administered, resulting in significant improvement in muscle strength and normalization of blood pressure. Our clinical observation illustrates a very unusual presentation of GBS, with the occurrence of arterial hypertension revealed by signs of intracranial hypertension, preceding the onset of acute flaccid paralysis by 20 days.

## Conclusions

In our observation, arterial hypertension was the only sign of dysautonomia, occurring 20 days before the onset of muscle weakness. No other cardiovascular, digestive, sudomotor, or urinary signs were present. This isolated presentation is rare and highlights that, in children, acute hypertension of unexplained etiology could represent an early sign of GBS, even in the absence of flaccid paralysis or other neurological manifestations at the time of its onset.
