# Correction: Isolated Arterial Hypertension as a Rare Early Manifestation of Guillain-Barré Syndrome: A Case Report

**DOI:** 10.7759/cureus.c437

**Published:** 2026-05-22

**Authors:** El Moufid Hajjar, Widade Kojmane

**Affiliations:** 1 Pediatric Emergency Department, Centre Hospitalier Universitaire Hassan II, Fès, MAR

This article has been corrected to include Widade Kojmane as an author. The authors deeply regret this oversight.

